# A Novel Missense Mutation in the Connexin30 Causes Nonsyndromic Hearing Loss

**DOI:** 10.1371/journal.pone.0021473

**Published:** 2011-06-24

**Authors:** Wen-Hung Wang, Yu-Fan Liu, Ching-Chyuan Su, Mao-Chang Su, Shuan-Yow Li, Jiann-Jou Yang

**Affiliations:** 1 Department of Otolaryngology, Chang Gung Memorial Hospital at Chiayi, Chang Gung University College of Medicine, Taiwan, Republic of China; 2 Department of Biomedical Sciences, Chung Shan Medical University, Taichung, Taiwan, Republic of China; 3 Department of Medical Research, Chung Shan Medical University Hospital, Taichung, Taiwan, Republic of China; 4 Department of Cardiovascular Surgery, Tian-Sheng Memorial Hospital, Tong Kang, Pin-Tong, Taiwan, Republic of China; 5 Department of Otorhinolaryngology-Head and Neck Surgery, Chung Shan Medical University Hospital, Taichung, Taiwan, Republic of China; 6 Institute of Medicine and Department of Speech Language Pathology and Audiology, Chung Shan Medical University, Taichung, Taiwan, Republic of China; Newcastle University, United Kingdom

## Abstract

Dysfunctional gap junctions caused by *GJB2* (CX26) and *GJB6* (CX30) mutations are implicated in nearly half of nonsyndromic hearing loss cases. A recent study identified a heterozygous mutation, c.119C>T (p.A40V), in the *GJB6* gene of patients with nonsyndromic hearing loss. However, the functional role of the mutation in hearing loss remains unclear. In this study, analyses of cell biology indicated that a p.A40V missense mutation of CX30 causes CX30 protein accumulation in the Golgi body rather than in the cytoplasmic membrane. The tet-on protein expression system was used for further study of mutant proteins in CX30 and CX30A40V co-expressions and in CX26 and CX30A40V co-expressions. The p.A40V missense mutation exerted a dominant negative effect on both normal CX30 and CX26, which impaired gap junction formation. Moreover, computer-assisted modeling suggested that this p.A40V mutation affects the intra molecular interaction in the hydrophobic core of Trp44, which significantly alters the efficiency of gap junction formation. These findings suggest that the p.A40V mutation in CX30 causes autosomal-dominant nonsyndromic hearing loss. These data provide a novel molecular explanation for the role of *GJB6* in hearing loss.

## Introduction

Hearing loss is a common sensory disorder in humans [1] and most auditory system dysfunctions resulting in hearing loss are genetically inherited [2]. The estimated incidence of congenital hearing loss is 1 in 1000 births, of which approximately 60% are attributable to genetic factors [3]–[4].

The inner ear consists of the hearing organ, the cochlea, are essential for hearing in the mammalian inner ears. The function of cochlea is dependent on tightly controlled ions, particularly K^+^ ions, main charge carrier for sensory transduction [5]. Ultrastructural studies have identified two gap junction networks, including that of the epithelial tissue and that of the connective tissue, in the mammalian cochlear duct and vestibular system [6], [7]. The gap junction system is the most likely pathway for cochlear K^+^ recirculation [6], [8], [9]. In the cochlea, connexin loss in gap junction complexes disrupt potassium recycling from the synapses at the base of hair cells through the supporting cells and fibroblasts back to the high potassium-containing endolymph of the cochlear duct. The resulting hearing loss is caused by local potassium intoxication of the Corti's organ [6].

Gap junction [GJ] channels mediate direct cell-to-cell communication by enabling intracellular transport of small biological molecules (<1 kDa), including electrolytes, second messengers and metabolites [10], [11]. The GJ channels have diverse functions, including electrical signal propagation, metabolic cooperation, growth control, spatial buffering of ions, and cellular differentiation [12]. The connexins (CXs/Cxs) are a family of proteins that form GJ channels in vertebrates. The human and mouse genomes contain twenty-one and twenty connexin genes, respectively [5]. The GJ channels formed by the head-to-head docking of two half channels, termed connexon, create hydrophilic pores across the membranes of two contact cells [13]. Each connexon is composed of six CX protein subunits [14]. The CXs/Cxs within a connexon may be similar (homomeric) or different (heteromeric), and the docking of two connexons may also be similar (homotypic junctions) or different (heterotypic junctions) [15]. Studies also indicate that CXs/Cxs can assemble into functional hexameric connexons in the ER membrane [16]. Subcellular fractionation studies and immunocolocalization analyses suggest that CXs/Cxs pass through the Golgi apparatus before reaching the plasma membrane [17]–[19].

Intercellular communication *via* gap junctions is crucial for auditory function. The important role of intercellular communication, particularly that between *GJB2* and *GJB6* (encoding CX26 and CX30), has been confirmed by evidence that certain connexin gene mutations cause sensorineural hearing loss [5], [20]–[23]. The *GJB6* gene (AJ005585) encodes a 261-amino acid protein. The human CX30 protein shares 93% homology with mouse Cx30 and 76% identity with human CX26. Northern blot, RT-PCR, and *in situ* hybridization analyses have revealed mouse Gjb6 expression in the trachea, thyroid, brain, and cochlea [21]. Immunocytochemistry studies of a 22-week-old human embryo have also revealed CX26 and CX30 in the lateral wall of the cochlea [24]. Recently published data suggest that no gross developmental defects in cochlear morphology are detected in both Cx26 and Cx30 mutant mice, but difference is the effect on the EP generation between the Cx26 and Cx30 mutant mice. In addition, the paper also indicate that Cx26 seems to be essential for survival and function of the organ of Corti, but is not required for its normal development. Therefore, two cochlear GJs, CX26 and CX30, were suggested do not play essential roles in cochlear development [25]. In some populations, *GJB6* mutations, including DFNB1 and DFNA3, reportedly cause hearing loss [21], [26]–[28]. The GJB6 deletion mutation del(GJB6-D13S1830) has been associated with hearing loss in nine countries [29]. However, the deletion mutation has not been detected in patients of Taiwan with nonsyndromic hearing loss in our previous study. In contrast, we identified one heterozygous missense variant, c.119C>T(p.A40V), and two heterozygous silent variants, c.261A>T (p.P87P) and c.396G>A (p.L132L) [22]. Presently, however, the functional alteration of CX30 caused by the variant *GJB6* gene remains unclear. To elucidate the pathogenic role of CX30 variant in nonsyndromic hearing loss, the functional properties of variant CX30 gap junctions must be clarified. This study therefore investigated the effect of the p.A40V (c.119C>T) variant on functional properties and on the subcellular localization of the heterozygous variant CX30 protein in HeLa cells and in tet-on HeLa cells.

## Materials and Methods

### Molecular cloning of wild-type and mutant *GJB6* gene

The mammalian expression vector pcDNA3.1-CT used in this study was constructed as previously described [30]–[31]. The open reading frames (ORFs) of *GJB6* were obtained from the genomic DNA using the PCR reaction. PCR was carried out with the following oligonucleotide primers: forward primer was *GJB6* F-HindIII 5′- atg**aagctt**atggattgggggacgctg-3′ and corresponded to nucleotides 1–18 of the human *GJB6* coding region; reverse primer was *GJB6* R-Xho1 5′- atg**ctcgag**gcgcttgggaaacctgtgattg -3′ and corresponded to nucleotides 762–783 of the human *GJB6* coding region. The PCR DNA product (801 bp) of the human *GJB6* coding region without stop coden was cloned into the pcDNA3.1-CT vector. Mutant CX30 gap junction proteins were obtained by performing oligonucleotide-directed mutagenesis using the Stratagene Quickchange site-directed mutagenesis kit (Stratagene,La Jolla,CA). The following oligonucleotide primers (mutated nucleotide is underlined) were used to prepare the mutant CX30A40V gene: CX30A40V sense 5′- ctagtggtggctg**t**ccaggaagtgtggg -3′; CX30A40V antisense 5′- cccacacttcctgg**a**cagccaccactag -3′. For fusion protein generation, cDNA sequences of the autofluorescent reporter proteins EGFP (pEGFPN1 vector; Clontech, Palo Alto, CA) and DsRed (pDsRedN1 vector; Clontech, Palo Alto, CA) were fused in-frame to the C-terminus of WT and mutant CX30A40V. The open reading frames (ORFs) of *GJB6* were obtained from the pcDNA3.1-CT clone after digestion with *Hind*III and *Sac*II, and then subcloned into the *Hind*III and *Sac*II restriction sites in vectors pEGFPN1 and pDsRedN1 (Clontech, Palo Alto, CA, USA). Further, the coding region of CX30WT and that of mutant CX30A40V were amplified from plasmids containing the CX30 cDNA (CX30WT-EGFP or CX30A40V-DsRed) using two pair primers containing recognition sequences 5′- *Sal*I (atg**gtcgac**tgatcagttatctagatccgg and 3′- *Not*I (atg**gcggccgc**gctagcgctaccggactc **) or 5′-*Nhe*I (agatcc**gctagc**gctaccg) and 3′-*EcoR*V (atg**gatatc**ccgctacaggaacaggtgg**), respectively, and Platinum Pfx DNA polymerase (Invitrogen, Carisbad, CA). Purified products were subcloned into the corresponding site of the bi-directional expression vector pBI (Clontech, Palo Alto, CA). The dideoxy DNA sequencing method, using a DNA Sequencing Kit (Applied Biosystems Corporation, Foster city, CA), an ABI Prism 3730 Genetic Analyzer (Applied Biosystems Corporation, Foster city, CA), and restriction digest were used to confirm the DNA sequence of all constructs.****

### Expression of CX30 gap junction proteins in HeLa cells

Human epitheloid cervix carcinoma cells (HeLa, ATCC CCL 2; American Type Culture Collection, Rockville, MD, USA) lacking the *GJIC* gene were used throughout this study. Cell lines were maintained in standard cell culture medium supplemented with 10% fetal bovine serum, 2 mM L-glutamine, and 50 units/mL penicillin–streptomycin. Cell cultures were maintained at 37°C in a humidified 5% CO_2_ incubator. The vectors pcDNA3.1-CT, pGFPN1, and pDsRedN1 containing the DNA fragment encoding the wild-type or mutant CX30 protein were transfected to HeLa cells using lipofectAMINE (Invitrogen Corporation, California, USA). The post-transfection cell line was used for the subsequent functional analyses.

### Transfection and expression of CX30WT, CX30A40V, CX30WT/CX30A40V and CX26WT/CX30A40V chimerae protein in tet-on HeLa cell line

The tet-on HeLa cell line deficient in the *GJIC* gene was purchased from BD Biosciences Clontech (Palo Alto, CA, USA) and maintained in Dulbecco's modified Eagle's medium, supplemented with 10% FBS (Gibco BRL, Gaithersburg, USA), 100 m“Insert>Symbol”g/ml G418, 100 U/ml penicillin, and 100 m“Insert>Symbol”g/ml streptomycin at 37°C in a moist atmosphere containing 5% CO2. Transfection was carried out using LipofectAMINE reagent (Invitrogen, Carlsbad, USA) according to the manufacturer's instructions. A ratio of 1 m“Insert>Symbol”g DNA vs. 2 m“Insert>Symbol”l LipofectAMINE 2000 was used for the tet-on HeLa cells. Cells were harvested at 24 h post-transfection and grown on a coverslip for 24 h at 37°C in a humidified 5% CO_2_ incubator. Then, tet-on HeLa cells were treated with 1 m“Insert>Symbol”g/ml doxycyclin (Dox) (Sigma-Aldrich Corporation, St. Louis, Mo) in cell culture medium to induce CX26WT, CX30WT, CX30A40V or both WT and mutant protein expression.

### Immunofluorescence staining of post-transfection HeLa and tet-on HeLa cells

Wild-type or mutant CX protein expression in HeLa and tet-on HeLa cells was analyzed by a direct fluorescent protein fusion method involving fusion of EGFP or DsRed to the C-terminal ends of the CX proteins. Briefly, post-transfection HeLa cells and tet-on HeLa cells grown on coverslips were fixed with 4% paraformaldehyde in 0.1 M PBS for 20 min and then rinsed three times in PBS. The tet-on HeLa cells grown on coverslips were exposed to Dox for 5 h prior to fixation. Then, the coverslips were immersed in 10% normal goat serum and 0.1% Triton X-100 for 15 min. The primary antisera and dilutions were as follows: mouse anti-pan-cadherin antibody at 1∶200 (anti-CH19; abcan) for cell membrane and mouse anti-Golgin-97 at 1∶200 (Invitrogen, Carisbad, CA) for Golgi apparatus. After incubation with primary antiserum at 4°C overnight, the cells were rinsed in PBS three times before adding Alexa Fluor 488 and/or Alexa Fluor 594 conjugated secondary antibodies (Invitrogen, Carisbad, CA). Endoplasmic Reticulum (ER) was stained with ER-Tracker® Blue-white DPX Probes at 1∶670 dilution (Invitrogen, Carisbad, CA) for 10 min at room temperature. The nuclei of cells were counterstained with 4′-6-Diamidino-2-phenylindole (DAPI; 2 m“Insert>Symbol”g/ml) or propidium iodide (PI; 1 mg/ml; 1∶400 dilution) for 5 min and rinsed with PBS. Mounted slides were visualized and photographed using a fluorescence microscope (Zeiss Axioplam, Oberkochen, Germany). All immunofluorescence cell experiments were performed more than five times and observed more than 200 pairs coupling cells per times with similar results in the study.

### Model building and Structural-based analysis

Although a detailed model of the crystalline structure of human CX30 is still unavailable, its closest homologous protein family member, human CX26 gene (amino acid identity 77%), which has been elucidated by crystallography (PDB entry 2ZW3) to a resolution of 3.5 Å, provides a suitable template [32]. Three-dimensional (3D) modeling of the human wild type and A40V mutation were performed using SWISS-MODEL, an automated homology modeling program. The homology-modeling server SWISS-MODEL is directly accessible at http://swissmodel.expasy.org/workspace/ [33], [34]. This study used the automatic modeling approach to apply the complete protein sequence of human Cx30, including its 261 amino acids and its mutation, which are available in NCBI GenBank (NP_006774.2) in FASTA format. For confirmation of energy criteria and for quality assessment of these protein models, anolea mean force potential was calculated to determine the energy of interactions indicated by the non-local environment of heavy atoms in protein chains [35]. Data obtained by the homology models were visualized using Accelrys™ ViewerLite version 4.2.

## Results

**Figure 1:**
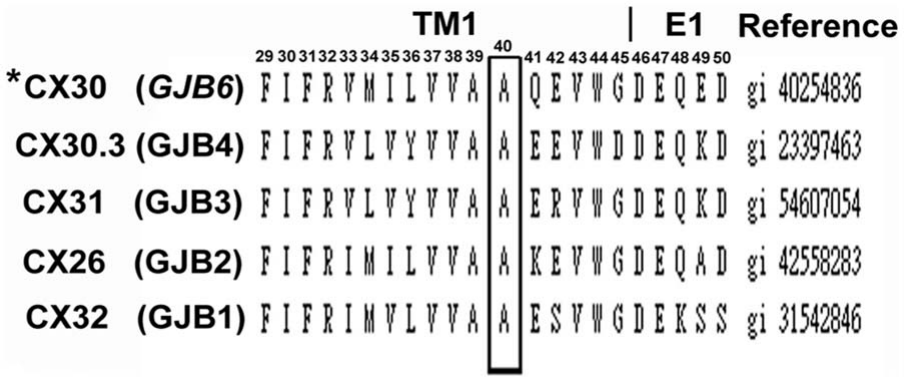
Alignment of amino acid sequences of the TM1 domain of CX30 proteins in members of the human b “Insert>Symbol”-group of CX family. The p.A40 residue, which was mutated in this study, is indicated in bold type. This alanine (A) at codon 40 is identical among members of human b “Insert>Symbol”-group of CX family. Human CX30 is indicated by an asterisk (*).

The topological model based on the UniProtKB/Swiss-Prot O95452 (CXB6_HUMAN) database showed that the CX30 protein subunit, like other CX proteins, contains a short cytoplasmic amino-terminal domain (NT; amino acid 1–22), four transmembrane domains TM; TM1 (amino acid 23∼45), TM2 (amino acid 76∼98), TM3 (amino acid 132∼154), and TM4 (amino acid 155∼192), which is separated by one cytoplasmic loop domain (CL; amino acid 99–131) and two extracellular loops (E1; amino acid 46–75 and E2; amino acid 155–192), and a carboxyl-terminal cytoplasmic domain (CT; amino acid 216–261). According to above predictive results, we extrapolated that the p.A40V substitution localized on the putative TM1 domain near the outer membrane (Figure S1).To clarify the roll of p.A40V, Biology WorkBench CLUSTAL W (1.81) Multiple Sequence Alignments (http://workbench.sdsc.edu/, San Diego Supercomputer Center) was used to compare amino acid sequences of Cx30 among the b “Insert>Symbol” group in the human CX family. The comparison revealed that p.A40 residue of CX30 protein was highly conserved among the human b “Insert>Symbol” group of the examined CX family members (Fig. 1). A basic ConSeq analysis system (http://conseq.tau.ac.il/) was also used to study amino acid sequences of CX30 in the CX gene family of all species. After the protein sequence of CX30 was deposited, the system automatically detected homologous sequences of CX30 and conducted multiple alignments. Of the 114 PSI-BLAST hits obtained by the system, 96 were unique sequences. In the next step, the system automatically calculated the 50 sequences with the lowest E-values. The calculation results revealed that p.A40 is highly conserved (Conseq score = 9) and buried in the CX protein (Figure S2). Therefore, mutation of the p.A40 residue may interfere with the normal function of CX30 protein and may play an important role in gap junction channel formation.

**Figure 2:**
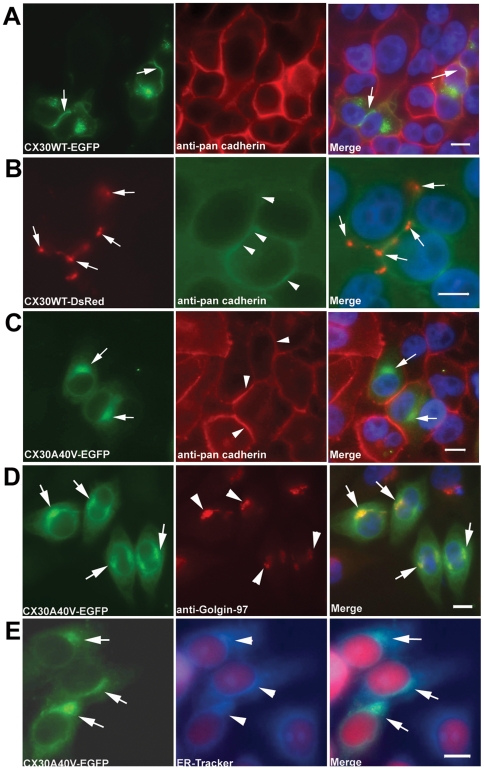
Analysis of CX30WT and CX30A40V expression in HeLa cells transiently transfected by immunocytochemistry. Fluorescence microscopy results for CX30WT-EGFP (A) and CX30WT-DsRed (B) HeLa cells showing expression of CX30 fusion protein in the plasma membranes using pan-cadherin antibody. The CX30A40V-EGFP (C) transfected HeLa cells show impaired trafficking and concentration near the nucleus. Intercellular localization of mutant CX30A40V protein. Photomicrographs of HeLa cells transiently transfected with CX30A40V-EGFP cDNA and immunostained for use as markers of Golgi apparatus (D) and ER (E). The staining results for mutant CX30A40V showed substantial co-localization in the Golgi apparatus and moderate co-localization with ER marker. Cells were counterstained with DAPI to highlight the nuclei. Arrows indicate CX30 protein localization. Scale bars: 10 m“Insert>Symbol”m.

To test the effects of p.A40V on the functional properties and subcellular localization of the CX30 protein, lipofection was used to transiently transfect gap junction-deficient HeLa cells with cDNA constructs of wild-type (CX30WT-GFP) or mutant CX30 (CX30A40V-GFP). In cells transiently expressing CX30WT-EGFP, gap junction plaque formation was indicated by WT CX30 expression localized to the cell membranes at points of contact between adjacent GFP-expressing cells. This membrane localization was confirmed by colocalization with pan-Cadherin (Fig. 2A). Similarly, CX30WT-DsRed was localized to the cell membrane (Fig. 2B). In contrast, the impaired trafficking of CX30A40V to the cell membrane resulted in cytoplasm concentrations near the nucleus (Fig. 2C). To identify the organelles in which the cytoplasm of the mutant p.A40V of CX30 had localized, this study then analyzed HeLa cells transfected with CX30A40V cDNA using immunostaining with markers for Golgi apparatus and endoplasmic reticulum (ER) (Fig. 2D and 2E). The analytical results indicated that most accumulation of p.A40V of CX30 mutant proteins were in the Golgi apparatus (Fig. 2D). Some proteins exhibited a reticular pattern and were co-localized with ER-tracker Blue-white DPX probe, an endoplasmic reticulum marker (Fig. 2E). In addition, these finding are consistent with our previous observation of subcellular localization of the CX30 protein using FlAsH-EDT_2_ labeling reagent and CX30 antibody (unpublished data; Figure S3).

**Figure 3:**
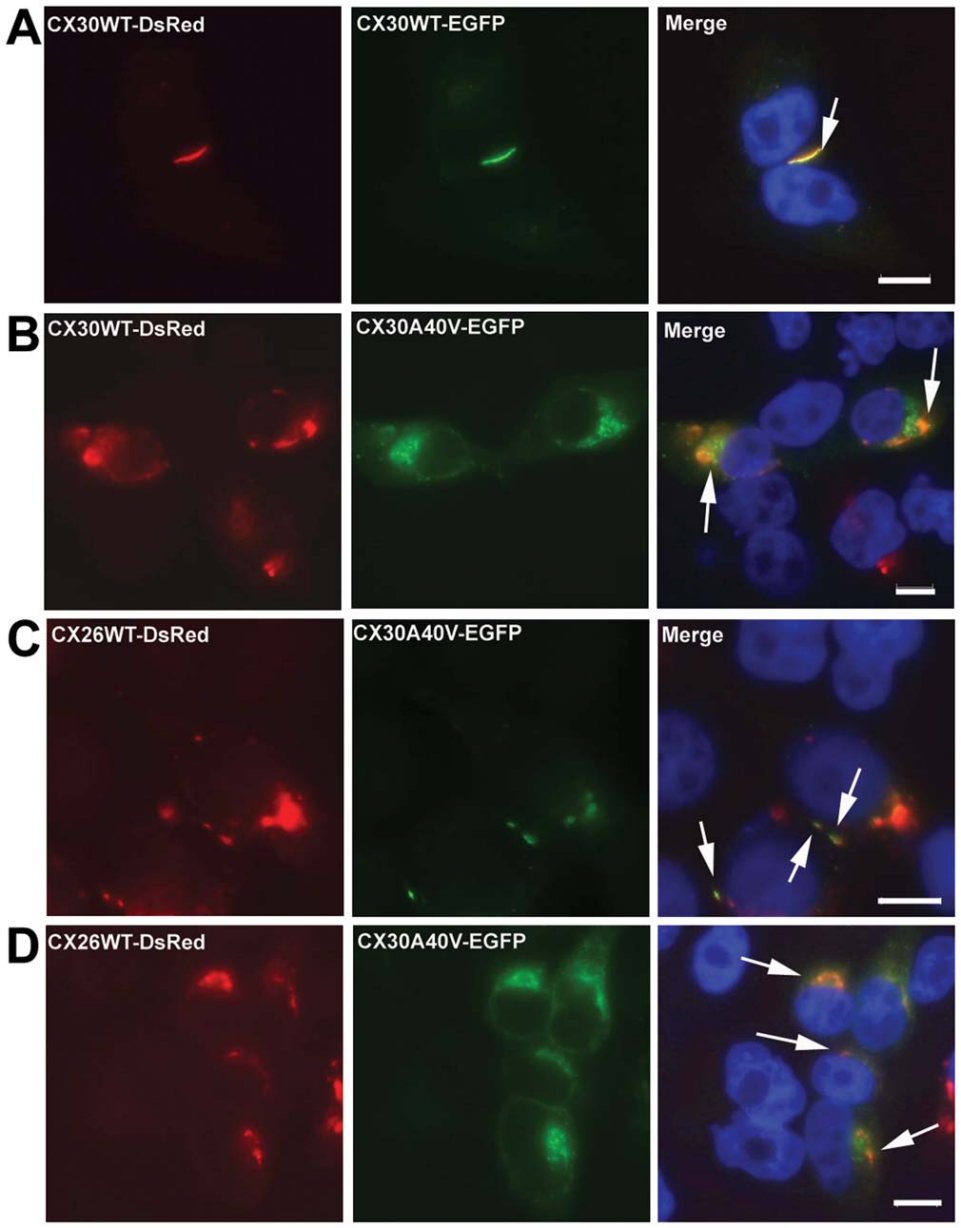
Co-expression of mutant proteins with CX30WT and CX26WT revealed by tet-on protein expression system. (A) Tet-on HeLa cells co-expressing CX30WT-DsRed and CX30WT-EGFP. Co-localization of the two proteins is visible at the plasma membrane. (B) Tet-on HeLa cells co-expressing CX30WT-DsRed and CX30A40V-EGFP. Co-localization of the two proteins is visible near the nucleus regions. (C) Tet-on HeLa cells co-expressing CX30WT-DsRed and Cx26WT-EGFP. Co-localization of the two proteins is visible at the plasma membrane. (D) Tet-on HeLa cells co-expressing CX26WT-DsRed and CX30A40V-EGFP. Co-localization of the two proteins is visible near the nucleus. Arrows indicate co-expressed proteins. Cells were counterstained with DAPI to highlight the nuclei. Scale bars: 10 m“Insert>Symbol”m.

Our previous study found that p.A40V of CX30 is a heterozygous mutation in patients with hearing loss [22]. Thus, co-expression studies were performed to test the effects of mutant proteins on CX30WT in tet-on HeLa cells. For this test, a bi-directional tet-on protein expression system was used with equal amounts of the two respective expression proteins. Cells expressing both CX30WT-DsRed and the CX30WT-EGFP protein exhibited co-assembly expression in the cell membrane of tet-on HeLa cells (Fig. 3A). Conversely, both CX30WT-RFP and the CX30A40V-EGFP mutant revealed expression patterns resembling those of p.A40V alone (Fig. 3B). Analytical results revealed co-assembly of CX30A40V mutant protein and CX30WT protein, resulting in impairment of CX30WT protein trafficking to the cell membrane.

Reports of CX30 and CX26 co-expression in the inner ear [2], [24], [36]–[37] prompted further tests of the effects of the mutant CX30A40V protein on CX26WT by using the tet-on protein expression system. Both CX30WT-DsRed and CX26WT-EGFP were colocalized at the points of contact between adjacent tet-on HeLa cells (Fig. 3C) which confirmed that both CX26 and CX30 can be trafficked to the same gap junction plaque. However, co-expression of CX30A40V-EGFP and Cx26WT-DsRed changed the localization of these CX26WT proteins from the cell membrane to the cytoplasm (Fig. 3D). Analytical results revealed co-assembly of CX30A40V mutant protein and CX26WT protein and impaired CX26WT protein trafficking to the cell membrane. We therefore hypothesized that p.A40V of CX30 has a dominant negative effect on CX30WT and CX26WT.

**Figure 4:**
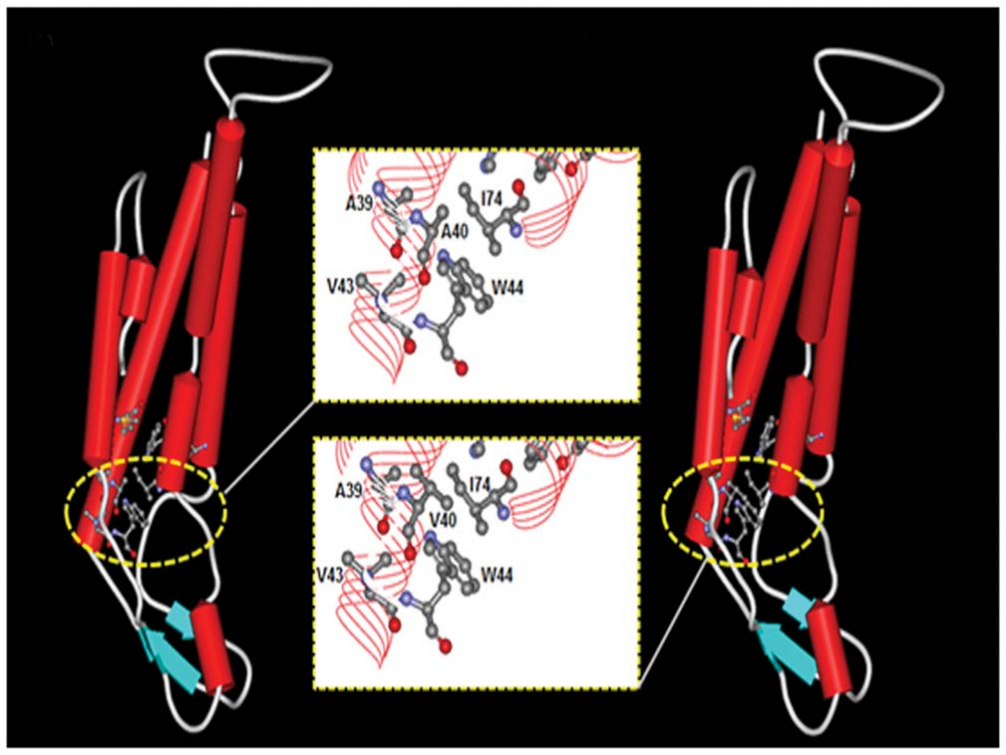
Bioinformatic estimation of three-dimensional (3D) structure of wildtype and p.A40V CX30 protein. Comparison of protein secondary structure architectures reveals the intra-molecular interactions between wild type (**A**) and A40V mutation (**B**) in monomeric human CX30. Secondary structures appear as red solid cylinders (α-helices), blue arrows (β-sheets) and white loops (turns). The yellow boxes indicate the close-up views of intra-molecular interactions focus on the extracellular part (E1) of the transmembrane region (TM2) in a similar orientation as in ball and stick representation. The highly conserved residues in this region, including Ala39, Ala40, Val43 and Ile74, contributed to the hydrophobic core around Trp44, which stabilized the Cx30 intra-molecular structure.

**Figure 5:**
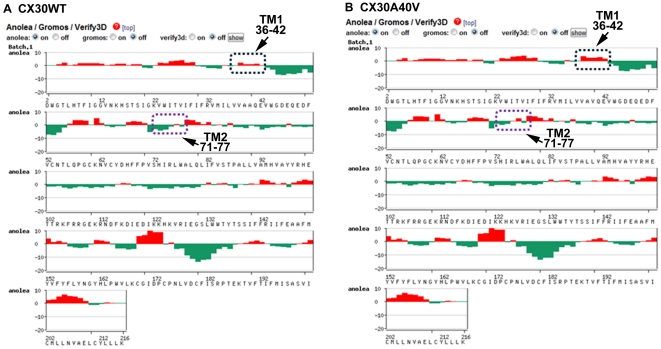
Plot outputs of Anolea mean force potential reported from SWISS-MODEL workspace. The graphs present the computer modeling results for wild-type (**A**) and p.A40V mutations (**B**) of the CX30 protein. Relative quality was evaluated by target-template similarity and modeling procedure. Non-local environments of each residue in the human Cx30 gene model (x-axis) were based on the template gene human Cx26 protein and on the percentage of energy change in all pairwise non-local interaction energy values between the template and modeling structure (represented by y-axis). Negative scores are plotted downwards and upwards to further validate the model by indicating the favorable and unfavorable energy environments, respectively. The arrows and boxes indicate that the two conserved regions contributing to the hydrophobic core around the key amino acid Trp44 were residues 36–42 located in the transmembrane 1 (TM1) region and residues 71–77 located in the TM2 region of the human Cx30 proteins, respectively.

To further study the protein-level mechanisms of the p.A40V mutation, a 3D model was constructed for a bioinformatic structural analysis. The analytical results found that the highly conserved residues in this region, including Ala39, Ala40, Val43 and Ile74, comprise a hydrophobic core around Trp44, which stabilizes the CX30 intra-molecular antiparallel organization of the helical structure between transmembrane domain 1 (TM1) and TM2 (Fig. 4). At codon 40 (p.A40V), therefore, a substitution of small side chain “alanine (A)” by long side chain “valine (V)” destroys the intra-molecular structure of the WT CX30 protein (Fig. 4B). Anolea mean force potential energy was also applied in the final models to compare the energy criteria with the energy change in mean force derived from mutation CX30 structure (Fig. 5). The force potential energy was changed at residues 36–42 and 71–77 located on TM1 and TM2. Based on these results, we suggest that p.A40 plays an important role in CX30 protein. Specifically, mutant p.A40V causes a loss of function in CX30 protein.

## Discussion

For auditory function, the important role of intercellular communication *via* gap junctions has been confirmed by findings that certain CX gene mutations, particularly those of *GJB2* and *GJB6*, cause hearing loss [38]. Studies of populations in Spain and [27] and elsewhere [29], [39] indicate that a 342-kb deletion in *GJB6* is the second most common cause of genetic deafness. A 309-kb deletion has also been reported in the *GJB6* gene of hearing loss patients [40]. In contrast, most studies agree that the proportion of point mutations is smaller than that of deletion mutations. One point mutation, c.14C>A (p.T5M), in the *GJB6* (CX30) is known to be involved nonsyndromic deafness by dominant inhibition of the activity of wild-type GJB6 in normal channels, underlining the critical function of GJB6 in normal hearing [21].Although none of the above mutations were observed in our previous study, we identified a missense heterozygous mutation, c.119C>T (p.A40V) of *GJB6*, in the patients with nonsyndromic hearing loss [22]. The biogenesis of gap junction intercellular communication channels involves a process leading to the formation of a hexameric connexon hemichannel embedded in the plasma membrane that docks with a connexon in the membrane of a neighbouring cell [41]. In addition, CX mutations can be divided into three categories: group 1 mutations, which only minimally affect normal protein function; group 2, which can alter communication, especially electrophysiological properties of gap junctions; and group 3, which are functionally deficient owing to major trafficking or targeting abnormalities of the connexins [42]. Therefore, p.A40V missense mutation in the *GJB6* gene may affect any one or more of these processes. In this article, we have been demonstrated that the missense mutation is belong to group 3 mutation that are functionally deficient owing to trafficking abnormalities.

This study found that the p.A40 residue of CX30 is located at the first transmembrane domain (TM1) and is identical among the human β group of the CX protein family and throughout the evolution of all species. The M1 domain may be a pore-forming domain [43] that is essential for transporting CX protein into plasma membrane [44]. The p.A39V, p.A39P and p.A40V of *GJB1* mutant have positions similar to those of the GJB6 p.A40V mutant located at the first transmembrane region and are retained in the ER (p.A39V, p.A39P) or in the Golgi (p.A40V) in HeLa cells [45], [46]. Disease-associated mutations in *GJB2* whose positions coincide with p.A40V of *GJB2* have also been detected in the keratitis ichthyosis deafness (KID) syndrome. [47]. Our data indicated that the p.A40V mutant protein of CX30 was retained in the Golgi apparatus of HeLa cells. Moreover, we observed significant inhibition of the functional activity of CX30-WT in HeLa cells when expressed in a manner mimicking a heterozygous genotype. Thus, p.A40V mutation has dominant negative effect on the function of WT CX30. Based on above findings, we suggested that the p.A40 amino acid likely plays a critical role in CX30, and as a result a mutation in this residue will lead to loss of function of the protein.

Mouse studies suggest that Cx26 (*Gjb2*) and Cx30 (*Gjb6*) are the two most abundantly expressed Cxs in the mouse cochlea [6], [37], [48]. In the inner ear, Cx26 and Cx30 share similar protein structures, expression distribution patterns, and colocalizations of two Cx isoforms within the gap junction plaques [48]–[50]. Some GJB2 gene mutations exert a selective transdominant inhibitory effect on the function of other connexins, including Cx30, that are preferentially expressed in the epidermis, skin appendages, the cornea and in deafness [36], [51]. Similarly, our co-expression study found that CX30-A40V mutant trafficking defects could not be rescued by co-expression with CX26-WT; instead, the p.A40V of the CX30 mutant causes loss of function in the WT CX26 protein. Therefore, we hypothesize that the p.A40V missense mutation has a trans-dominant negative effect. To date, no studies have shown that Cx30 mutations have trans-dominant inhibitory effects on the function of other connexins. This co-expression study is the first evidence that p.A40V of the CX30 mutation has a trans-dominant negative effect on CX26.

Improved 3D characterization of protein structures is needed to clarify the functions of proteins and their disease formation relationships [33], [52]. High-resolution characterization of proteins can be provided by either experimental methods such as X-ray crystallography and nuclear magnetic resonance or by computational analysis [52]. However, the number of known protein sequences far exceeds that of experimentally solved protein structures. In the absence of crystallographic structures, a variety of advanced homology modeling methods have been developed, which can provide reliable models of proteins that share 30% or more sequence identity between known structures and target proteins [33], [34]. Homology modeling is currently the most accurate computational method to generate reliable structural models and is routinely used in many biological applications [34]. Homology models of a protein of interest is a valuable tool for interpreting sequence variations and for designing mutagenesis experiments to elucidate the biological functions of proteins [53], [54]. The crystalline structure of the gap junction channel formed by human CX26 has also been reported recently. The paper was shown that eight key residues (V43, W44, A39, A40, I74, W77, F154 and M195) have intra-protomer interactions within two hydrophobic cores of CX26 protein and stabilize the CX26 connexin structure [32]. In the clinical data, mutations of these residues are associated with deafness and skin diseases [55]. The pore-lining residues of CX26 at the TM1/E1 boundary are K41, E42 and G45 [32]. In the study, we compared above eight key residues of CX26 at two hydrophobic cores with CX30 results in identical between CX26 and CX30. Further, using a simulated model with human Cx26 protein (PDB ID is 2ZW3) as a template and an automatic SWISS-MODEL homology modeling program, this study found that the A40 of CX30 in the first hydrophobic core, not in the pore region, around Trp44 stabilizes the CX30 intra-molecular antiparallel helical structure between transmembrane segment 1 (TM1) and TM2. In previous paper indicated that most of the residues involved in intra-protomer interactions are conserved within the connexin family and mutations probably interfere with the proper folding and/or oligomerization of connexins, thus resulting in defective channels [32]. Therefore, our results of prediction obtained by homology modeling methods can elucidate functional deficiencies observed in immunofluorescence experiments, which is p.A40V missense mutation of CX30 caused CX30 protein accumulation in the Golgi body rather than in the cytoplasmic membrane.

Recently, there are reported to indicate that the mutations, p.A40V of CX26, which is positioned near the TM1/E1 boundary and are associated with the keratitis ichthyosis deafness (KID) syndrome. The mutants have been reported to form hemichannels that open aberrantly, leading to “leaky” cell membranes and show significantly impaired regulation by extracellular Ca^2+^, increasing the likelihood of aberrant hemichannel opening [56]. However, our result found that the dominant effect exerted by CX30 p.A40V mutation, which is dramatically different with p.A40V of CX26. We compared pore-lining residues of CX26 at the TM1/E1 boundary with CX30 results in the residue of p.41 from K (Lys) into Q (Gln) between CX26 and CX30. The residue of K41 creates a narrowed part of the pore with the diameter of about 17A° and is unique to CX26, generating a more positively charged environment between the funnel and the following negatively charged part of the solute pathway [32]. In addition, Cx26 and Cx30 share the same pattern of expression in the mice cochlea and form homotypic or heterotypic channels. The trafficking and assembly features of CX30 are also similar to those of CX26 [50]. Base on the above results, it will help us to explain the dominant negative effect of WT CX26 by CX30 p.A40V, which is different molecular machinery in only CX26 p.A40V mutation. However, these are prediction results and we only build the “single unit” of the Cx30 3D structure model based on the CX26 crystal structure [32] in the study. Simultaneously, the permeation pathway of a gap junction channel consists of an intracellular channel entrance, a pore funnel and an extracellular cavity. Therefore, further X-ray crystallographic studies are needed to clarify how this mutation affects the crystalline structure of wild-type and mutant CX30 or CX26 proteins.

## Supporting Information

Figure S1 **Schematic representation of the domain structure of the CX30 protein with indication of known variants.** The black star and *arrows indi*cate the c.119C>T (p.A40V) variant in CX30. M1-4: transmembrane domains; E1-2: extracellular domains; CL: cytoplasmic linking domain; N: N-terminal domain; C: C-terminal domain. [This figure was modified from Figure 4 in the text entitle “Attachment, polarity and communication characteristics of bone cells” by Ilvesaro J. (2001).]
(TIF)

Figure S2 **ConSeq predictions demonstrated on human CX30 [SWISS-PROT: O95452 (CXB6_Human)], using 50 homologues obtained from the Pfam database (family code: PF00029).** The sequence of the CX30 protein is displayed with the evolutionary rates at each site colour-coded onto it (see legend). The residues of the Cx26 sequence are numbered starting from 1. The first row below the sequence lists the predicted burial status of the site (i.e. “b”—buried versus “e”—exposed). The second row indicates residues predicted to be structurally and functionally important: “s” and “f”, respectively. Vertical arrows indicate amino acid codons (p.A40).
(TIF)

Figure S3 **Expression analysis of CX30WT and CX30A40V in transiently transfected HeLa cells using the FlAsH™-EDT_2_ Labeling Kit (A) and anti-CX30 antibody (B, C).** (A, B) Fluorescence microscopy of CX30WT HeLa cells shows expression of the wild-type protein in the plasma membranes. (C) In contrast, CX30A40V transfected HeLa cells show expression of the mutated protein near the nucleus. Arrows indicate expression of CX30 protein. Scale bars: 10 µm.
(TIF)
