# Supplementary figures and images for: A Novel Missense Mutation in the Connexin30 Causes Nonsyndromic Hearing Loss
Source: PLoS One. 2011 Jun 24;6(6):e21473. doi: 10.1371/journal.pone.0021473 (PMC3123352; doi:10.1371/journal.pone.0021473)

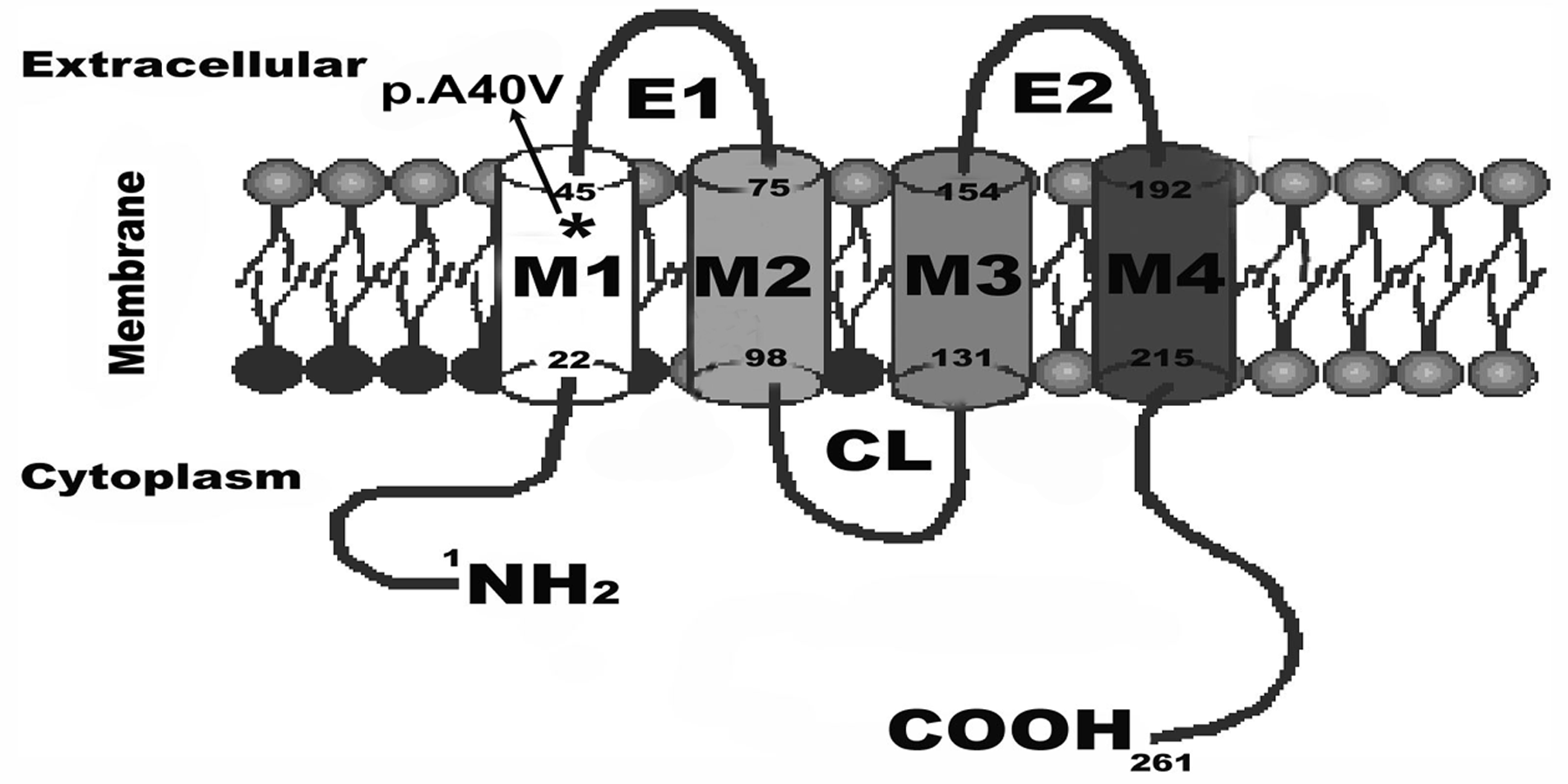

Supplement: Figure S1 — Schematic representation of the domain structure of the CX30 protein with indication of known variants. The black star and arrows indicate the c.119C>T (p.A40V) variant in CX30. M1-4: transmembrane domains; E1-2: extracellular domains; CL: cytoplasmic linking domain; N: N-terminal domain; C: C-terminal domain. [This figure was modified from Figure 4 in the text entitle “Attachment, polarity and communication characteristics of bone cells” by Ilvesaro J. (2001).] (TIF) [file pone.0021473.s001.tif]

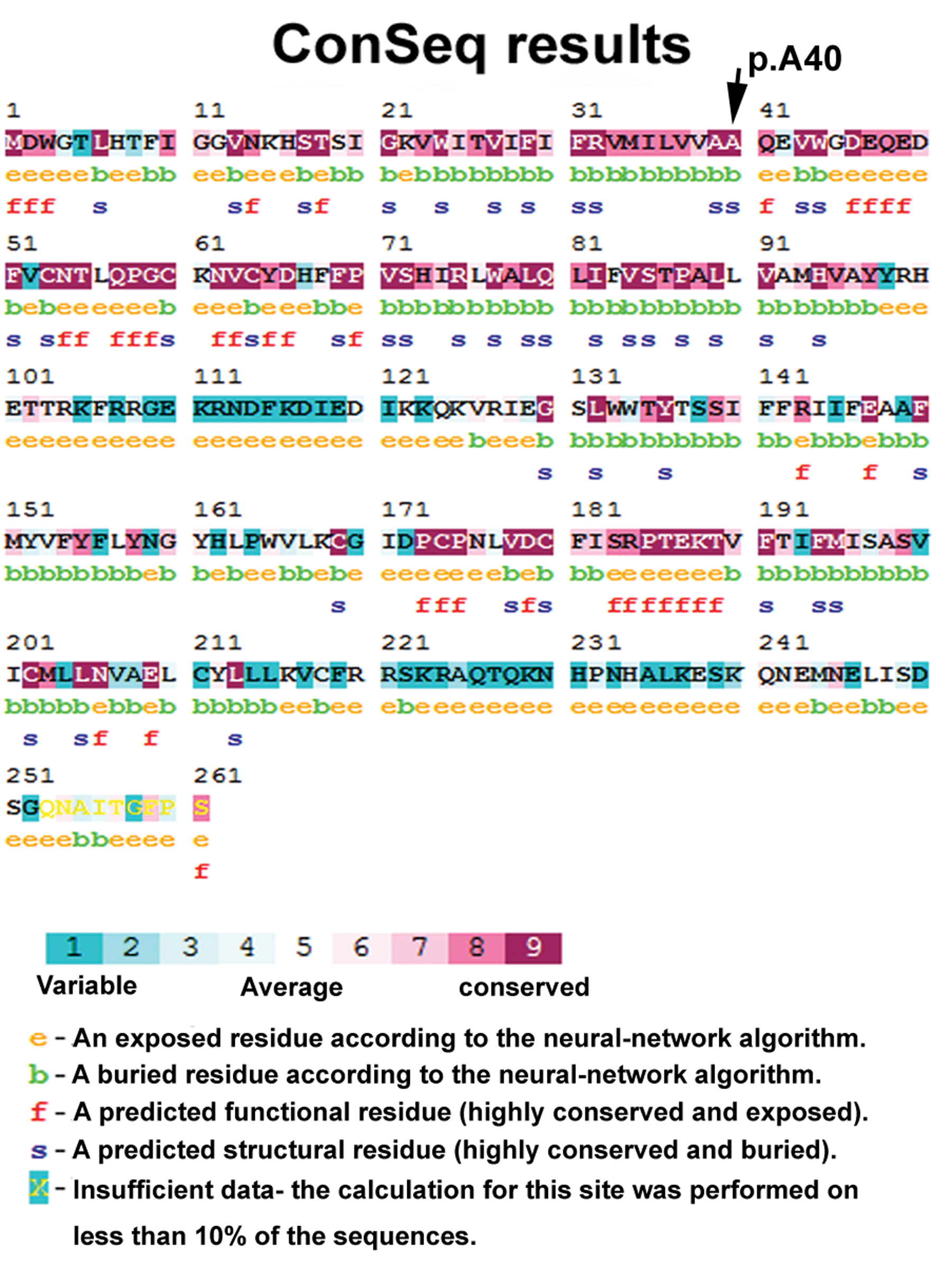

Supplement: Figure S2 — ConSeq predictions demonstrated on human CX30 [SWISS-PROT: O95452 (CXB6_Human)], using 50 homologues obtained from the Pfam database (family code: PF00029). The sequence of the CX30 protein is displayed with the evolutionary rates at each site colour-coded onto it (see legend). The residues of the Cx26 sequence are numbered starting from 1. The first row below the sequence lists the predicted burial status of the site (i.e. “b”—buried versus “e”—exposed). The second row indicates residues predicted to be structurally and functionally important: “s” and “f”, respectively. Vertical arrows indicate amino acid codons (p.A40). (TIF) [file pone.0021473.s002.tif]

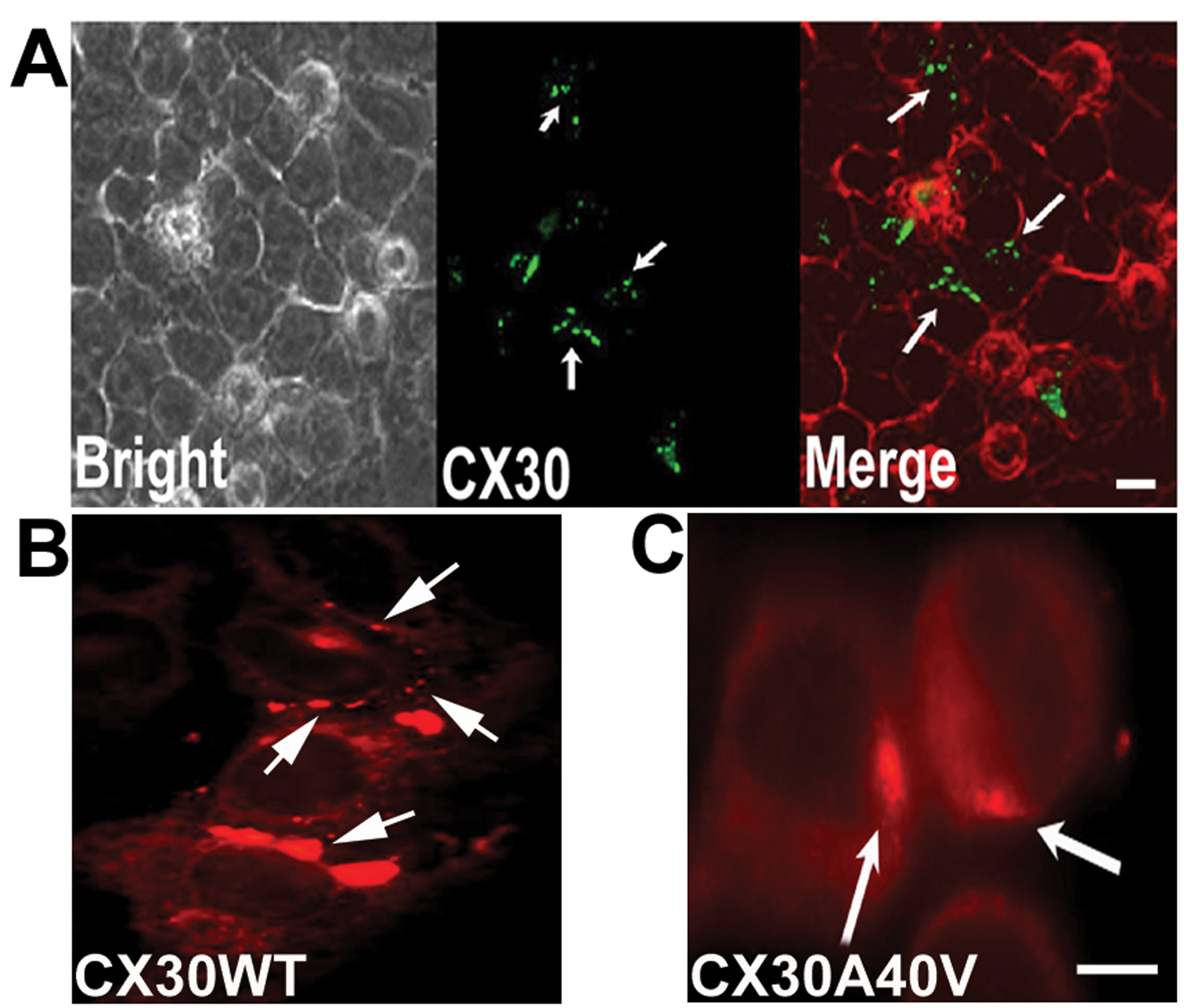

Supplement: Figure S3 — Expression analysis of CX30WT and CX30A40V in transiently transfected HeLa cells using the FlAsH™-EDT2 Labeling Kit (A) and anti-CX30 antibody (B, C). (A, B) Fluorescence microscopy of CX30WT HeLa cells shows expression of the wild-type protein in the plasma membranes. (C) In contrast, CX30A40V transfected HeLa cells show expression of the mutated protein near the nucleus. Arrows indicate expression of CX30 protein. Scale bars: 10 µm. (TIF) [file pone.0021473.s003.tif]
